# Insights into the leveraging of GABAergic signaling in cancer therapy

**DOI:** 10.1002/cam4.6102

**Published:** 2023-05-18

**Authors:** Tian‐Jiao Li, Jian Jiang, Ya‐Ling Tang, Xin‐Hua Liang

**Affiliations:** ^1^ State Key Laboratory of Oral Diseases, National Clinical Research Center for Oral Diseases, Department of Oral and Maxillofacial Surgery West China Hospital of Stomatology, Sichuan University Chengdu China; ^2^ Department of Head and Neck Surgery, Sichuan Cancer Center School of Medicine, Sichuan Cancer Hospital & Institute, University of Electronic Science and Technology of China Chengdu China; ^3^ State Key Laboratory of Oral Diseases, National Clinical Research Center for Oral Diseases, Department of Oral Pathology West China Hospital of Stomatology, Sichuan University Chengdu China

**Keywords:** cancer, carcinogenesis, GABA, GABA receptor, neurotransmitter

## Abstract

Gamma‐aminobutyric acid (GABA) is the main inhibitory neurotransmitter in the brain of adult mammals. Several studies have demonstrated that the GABAergic system may regulate tumor development via GABA receptors, downstream cyclic adenosine monophosphate (cAMP) pathway, epithelial growth factor receptor (EGFR) pathway, AKT pathway, mitogen‐activated protein kinase (MAPK) or extracellular signal‐related kinases (ERK) pathway, and matrix metalloproteinase (MMP) pathway, although the exact mechanism is unclear. Pioneering studies reported that GABA signaling exists and functions in the cancer microenvironment and has an immunosuppressive effect that contributes to metastasis and colonization. This article reviews the molecular structures and biological functions of GABAergic components correlated with carcinogenesis, the mechanisms underlying GABAergic signaling that manipulate the proliferation and invasion of cancer cells, and the potential GABA receptor agonists and antagonists for cancer therapy. These molecules may provide an avenue for the development of specific pharmacological components to prevent the growth and metastasis of various cancers.

## 1 INTRODUCTION

Gamma‐aminobutyric acid (GABA) is the principal inhibitory neurotransmitter in the central nervous system (CNS) that acts via the activation of specific GABA receptors. GABA is synthesized from glutamate, glutamine, and glucose by the glutamic acid decarboxylase enzymes GAD65 and GAD67 in the CNS.^1^, ^2^ GABA is activated by combining ionotropic GABA_A_ receptors or metabotropic GABA_B_ receptors^3^ and controls the proliferation, differentiation, migration, and death of cells during nervous system development.^4^ Recent studies revealed that GABA and its receptors also express and have diverse physiological functions in the development and maturation of nonneuronal peripheral tissue,^5^ including the palate,^6^, ^7^ lungs,^8^, ^9^ digestive tract,^10^, ^11^ pancreas,^12^ liver,^13^ testicular cells,^14^, ^15^ and even stem cells.^16^

Neurotransmitter systems contribute to multiple malignancies. Increasing reports demonstrated that the expression of GAD, GABA, and GABA receptors was significantly higher in cancers, including colon cancer, lung cancer, and gastric cancer than in normal tissues.^17^, ^18^, ^19^, ^20^, ^21^, ^22^, ^23^ Cancer cell proliferation and invasion are regulated by the binding of GABA to GABA_A_ or GABA_B_ receptors, thus, opening new avenues to develop pharmacological agonists and antagonists for cancer therapy. This review describes the potential mechanisms of GABAergic signaling in cancer development and progression and the possible treatment of cancer using GABA receptor‐associated drugs.

## 2 THE GABAergic SYSTEM

**FIGURE 1:**
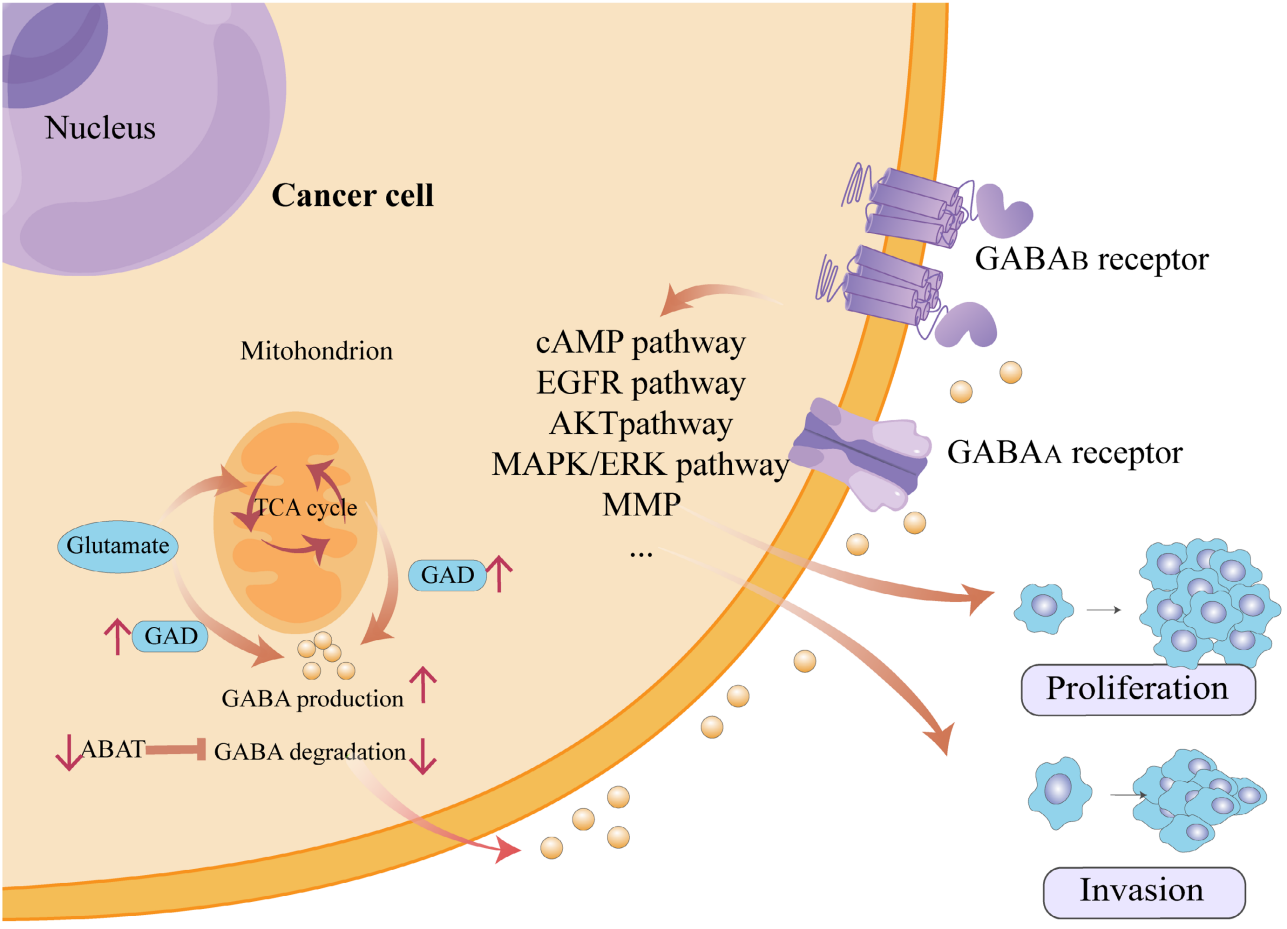
The schematic diagram of GABA synthesis and the potential mechanisms of GABAergic components in cancer proliferation and invasion. In cancer cells, GABA is formed from glutamate directly or via TCA cycle metabolism, and high level of GABA in the tumor microenvironment due to increased GABA production from GAD and decreased GABA degradation by ABAT. Then the synthesized GABA activates the GABA receptors by combining with the extracellular binding domains of GABA_A_ receptors or GABA_B_ receptors to regulate cancer cell proliferation and/or invasion. ABAT, 4‐aminobutyrate aminotransferase; cAMP; cyclic adenosine monophosphate; EGFR, epithelial growth factor receptor; GABA, gamma‐aminobutyric acid; GAD, glutamic acid decarboxylase enzyme; MAPK/ERK, mitogen‐activated protein kinase/extracellular signal‐related kinase; MMP, matrix metalloproteinase; TCA, tricarboxylic acid.

The GABAergic system functions as an inhibitory neurotransmitter in the CNS and mainly contains GABA, GABA transporters, GABA receptors, and GABAergic neurons.^24^ In GABAergic neurons, GABA can be directly synthesized by the decarboxylation of glutamate in the cytosol.^25^ Alternatively, GABA can be indirectly formed from glutamate by bypassing the tricarboxylic acid (TCA) cycle. This process is called the GABA shunt, a closed‐loop system in charge of the production and conservation of GABA supply^25^ (Figure 1). GABA synthesis is mediated by two GAD isoforms, namely, GAD65 and GAD67. GAD65 mainly participates in GABAergic synaptic transmission and plasticity, whereas GAD67 manipulates metabolic GABA synthesis.^26^ Notably, GAD67 is upregulated in tumors and contributes to cancer progression, whereas GAD65 remains unreported.^27^ GAD67 facilitates tumor progression as it is overexpressed in tumor tissues with greater proliferative and invasive potential.^28^, ^29^, ^30^ Moreover, high DNA hypermethylation, mRNA expression, and protein expression of GAD67 have been correlated with advanced tumor status, which makes it a potentially important prognostic indicator of poor outcomes in several cancer types.^27^, ^31^, ^32^, ^33^ The GABA transaminase 4‐aminobutyrate aminotransferase (ABAT) catabolizes GABA into succinic semialdehyde. Reduced expression of ABAT in some cancer cells may promote cancer cell proliferation and migration due to the accumulation of GABA.^34^, ^35^, ^36^ Therefore, the inhibition of GAD67 expression and promotion of ABAT expression may reduce cancer progression.

**FIGURE 2:**
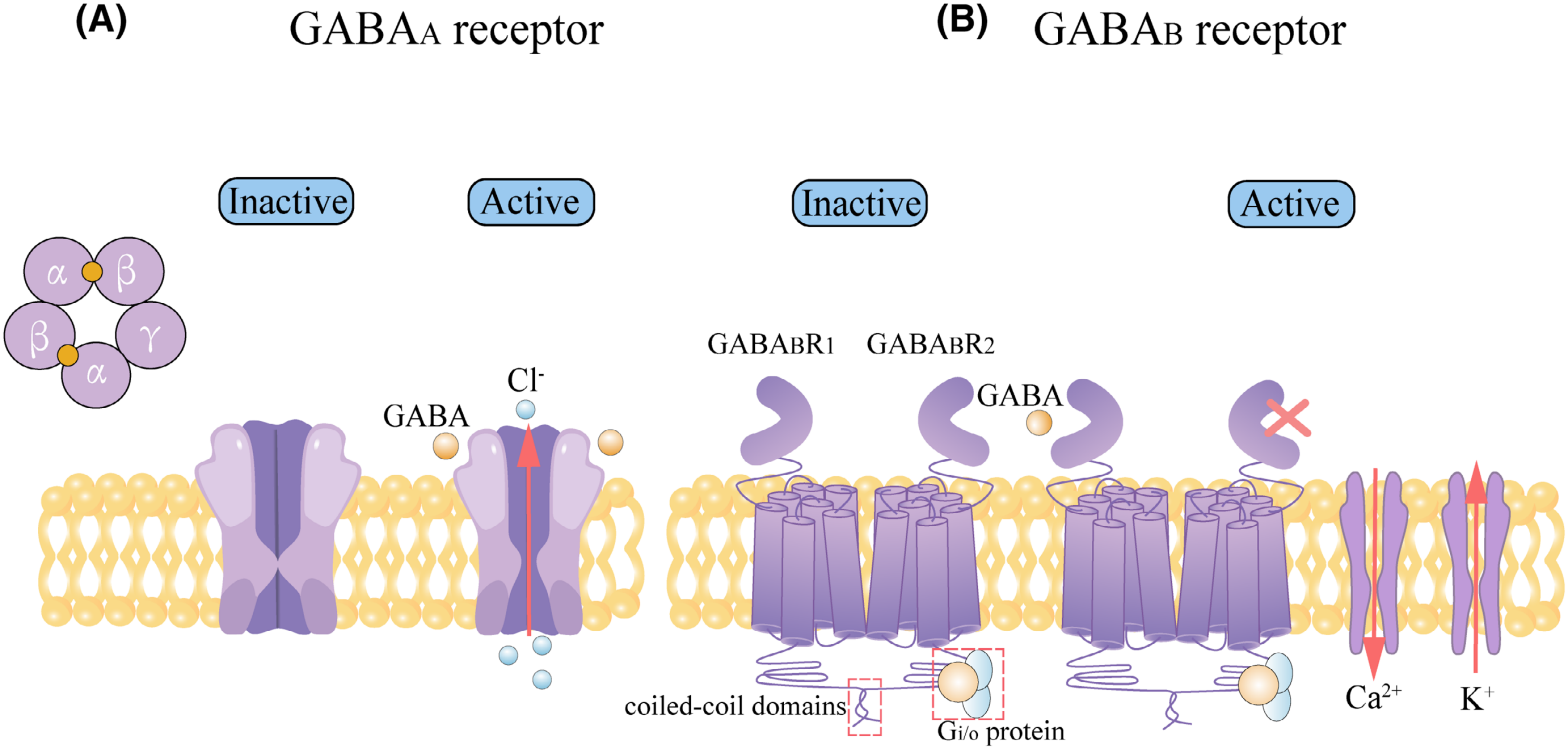
GABA_A_ receptor and GABA_B_ receptor molecular and functional structure. (A) GABA_A_R is a pentameric ligand‐gated ion chloride channels consisting of five transmembrane subunits. The most abundant GABA_A_R subtype consists of 2α, 2β, and 1γ subunits. GABA_A_R is activated by binding with GABA to open Cl^−^ channel. (B) GABA_B_R is a heterodimeric G‐protein‐coupled receptor composed of the GABA_B_R_1_ subunit and GABA_B_R_2_ subunit. The subunits interact with each other via intracellular coiled‐coil domains near the C terminus. The intracellular loops of GABA_B_R_2_ are coupled to Gi/o‐type G‐proteins. The extracellular domain of GABA_B_R_1_ can bind GABA, whereas the extracellular domain of GABA_B_R_2_ has no ligand‐binding activity. GABA_B_R is activated by binding with GABA to regulate K^+^ channels and Ca^2+^ channels. GABA_A_R, GABA_A_ receptor; GABA_B_R, GABA_B_ receptor; GABA_B_R_1_, GABA_B_ receptor 1; GABA_B_R_2_, GABA_B_ receptor 2.

GABA functions via two types of receptors, namely, ionotropic GABA_A_ and GABA_C_ receptors and metabotropic GABA_B_ receptors, which have different structures and functions (Figure 2). GABA_A_ receptors (GABA_A_R) are members of ligand‐gated chloride channels families consisting of the assembly of multiple subunit subtypes (α1–6, β1–3, γ1–3, δ, ϵ, θ, π, ρ1–3) into a pentamer. The pharmacological and functional properties of GABA_A_R channels closely depend on their subunit composition. The most abundant GABA_A_R subtype consists of two α, two β, and one γ subunits.^37^ GABA_A_R is activated by binding with GABA to open the Cl^−^ channel, subsequently inducing the hyperpolarization of the postsynaptic membrane. Contrary to GABA‐binding sites, GABA_A_R possesses several allosteric binding sites for anxiolytic, hypnotic, anesthetic, and anticonvulsant drugs.^38^ For instance, bicuculline and picrotoxin are antagonists of GABA_A_R, whereas propofol and benzodiazepines are its agonists.^39^, ^40^, ^41^ GABA_B_ receptors (GABA_B_R) are members of the family of heterodimeric G‐protein‐coupled receptors, which are composed of two main GABA_B_R subunits termed GABA_B_ receptor 1 (GABA_B_R_1_) subunit and GABA_B_R_2_ subunit.^42^ Each subunit contains a C‐terminal intracellular domain, an N‐terminal extracellular domain, a heptahelical transmembrane domain, and an extracellular Venus flytrap domain. The subunits interact with each other via an intracellular coiled‐coil domain near the C terminus, which can mask the reticulum retention signal. GABA_B_R can be activated by GABA to regulate Ca^2+^ channels and K^+^ channels.^43^, ^44^ GABA activates serious proteins connecting with the C‐termini of GABA_B_R by binding with the extracellular domain of GABA_B_R_1_, whereas the extracellular domain of GABA_B_R_2_ has no ligand‐binding activity. Some allosteric modulators, such as CGP7930, can bind the transmembrane domain of GABA_B_R_2_ to modulate the binding affinity of the ligand with GABA_B_R_1_, as well as the efficacy of signal transduction after binding.^45^ However, the mechanisms governing downstream signaling remain unclear.

All components of the GABAergic system have also been reported to be highly expressed in peripheral organs such as the liver,^46^ pancreas,^47^, ^48^ prostate,^49^ kidneys,^50^ intestines,^51^ testes,^52^ and ovaries,^53^ thus highlighting the pivotal effect of the GABAergic system in human development.

## 3 GABAergic SIGNALING IN CANCER

### 3.1 The effects of GABA and its receptors in different cancers

Recent studies have demonstrated that GABA exerts crucial regulatory effects on various types of cancers by binding with its receptors. In most cases, the expression levels of GABA and its receptors showed significant changes in tumor tissues compared with normal tissues, with GABA affecting cellular proliferation through GABA_A_R and cellular invasion through GABA_B_R. However, it is unclear as to whether GABAergic signaling could serve a positive or negative role in the regulation of cancer cell behavior (Table 1). GABAergic signaling may have different functions that depend on the origin of the tumor and the type of receptor subunits, which underscore the need to understand the GABA crosstalk in cancer.

**TABLE 1:** The expression, effect, and pathways of GABA and GABA receptors in different cancers.

Region	Cancer	Expression	Cancer development	Mechanisms	Studies
Brain	Glioblastoma	GABA_A_R_1_ ↑	Negative	GABA_A_R, miR‐155	104, 105
Head and neck	Oral cancer	GAD1↑, GABA_A_R_P_↑	Positive	GAD1, GABA_A_R_P_	56, 81
	Nasopharyngeal carcinoma	GAD1↑	Positive	GAD1	^31^
Lung	NSCLC	GABA↓, GABA_A_R_A3_↑, GABA_A_R_E_↑, GABA_B_R_2_↑	Negative	GABA_B_R, cAMP	57, 106
	PAC	GABA↓	Negative	GABA_B_R, cAMP, Erk1/2	^58^
Breast	Breast cancer	GABA↓	Negative	GABA_A_R	66, 99
Stomach	Gastric cancer	GABA↑, GAD65(+), GABA_A_R (+), GABA_B_R (−)	Positive	GABA_A_R, MAPK/ERK	^23^
Liver	Hepatocellular carcinoma (HCC)	GABA_A_R_β3↓_	Negative	GABA_A_R_β3_, GABA_B_R cAMP, ERK1/2	62, 63, 96
		GABA_A_R_Q_↑, GABA_A_R_A3_↑	Positive	GABA_A_R	17, 61
	Cholangiocarcinoma	GABA_A_R_β3_ (+), GABA_B_R_1_(+), GABA_C_R (+)	Negative	GABAR, cAMP, MAPK/ERK	^60^
Colon	Colon cancer	GABAR (+)	Negative	GABAR, cAMP, MMP	19, 59
Pancreas	PDAC	GABA↓	Negative	GABA_B_R, cAMP, ERK1/2	^64^
		GABA_A_R_P_↑	Positive	GABA_A_R_P_, MAPK/ERK	^30^
Prostate	Prostate cancer	GABA↑, GAD67↑, GABA_A_R↑	Positive	GABA_A_R and GABA_B_R, MMP	18, 22
Ovarian	Ovarian cancer	GABA↑	Positive	GABA_A_R_P_	55, 107
Thyroid	Thyroid tumors	GABA_A_R_B2_↑, GABA_A_R_A2_ (+), GABA_B_R_2_↑	Positive	GABA_A_R_β2_, GABA_A_R_α2_, GABA_B_R_2_	^108^

Abbreviations: GAD, glutamic acid decarboxylase enzymes; GABAR, GABA receptor; GABA_A_R_1_, GABA_A_ receptor 1; GABA_A_R_A2_, GABA_A_ receptor subunit α2; GABA_A_R_A3_, GABA_A_ receptor subunit α3; GABA_A_R_E_, GABA_A_ receptor subunit ε; GABA_B_R_2_, GABA_B_ receptor subunit 2; GABA_A_R_Q_, GABA_A_ receptor subunit θ; GABA_A_R_P_, GABA_A_ receptor subunit π; GABA_A_R_β2_, GABA_A_ receptor subunit β2; GABA_A_R_β3_, GABA_A_ receptor subunit β3.

#### 3.1.1 The effects of GABA on cancers

The GABA pathway plays a dual role and can be differentially regulated in different cell populations. GABA promotes tumor development in prostate cancer,^18^, ^22^, ^54^ gastric cancer,^23^ ovarian cancer,^55^ and oral squamous cell carcinoma (OSCC).^56^ In contrast, GABA exerts inhibitory effects on non‐small cell lung cancer (NSCLC),^57^ pulmonary adenocarcinoma (PAC),^58^ colon cancer,^59^ and cholangiocarcinoma.^60^ Notably, GABA and its receptors have a dual function in hepatocellular carcinoma (HCC),^17^, ^61^, ^62^, ^63^ pancreatic ductal adenocarcinoma (PDAC),^30^, ^64^ and breast cancer.^65^, ^66^, ^67^, ^68^ In HCC, Li et al^17^ found that GABA promotes the proliferation of hepatoma cells through the GABA_A_R θ subunit (GABA_A_R_Q_), while Yan et al^61^ found that this effect occurs through the GABA_A_R α3 subunit (GABA_A_R_A3_). However, other subunits of GABA receptors may play an opposite role. Minuk et al^62^ reported that the expression of hepatic GABA_A_R β3 subunit (GABA_A_R_B3_) was downregulated in human HCC, and its restoration can attenuate tumor growth in nude mice. In PDAC, Takehara et al^30^ observed that GABA promoted the proliferation of PDAC cells through the GABA_A_R π subunit (GABA_A_R_P_), whereas Schuller et al^64^ demonstrated that GABA inhibited PDAC cell proliferation and migration by inhibiting the isoproterenol‐stimulated pathway, which may be suppressed in human PDAC tissue. In breast cancer, GABA promotes the proliferation and invasion of breast cancer cells through GABA_B_R_1e_, GABA_A_R_A3,_ ^68^ and GABA_A_R_P._ ^67^ However, Drell et al^66^ reported that GABA inhibited the norepinephrine‐induced migration of breast cancer cells, which may be mediated by GABA_B_R, as confirmed by the use of a specific GABA_B_R agonist, baclofen. The dichotomous role of GABA on cancers may be attributed to the diverse subunits of GABA receptors.

#### 3.1.2 The effect of GABA_A_ receptors on cancers

Several studies have reported that GABA can promote cell proliferation through the GABA_A_R in cancers such as HCC,^17^, ^61^ prostate cancer,^22^, ^54^ gastric cancer,^23^ breast cancer,^67^, ^68^ PDAC,^30^ and OSCC,^56^ and inhibit cell proliferation through the GABA_A_R in cancers such as HCC^62^ and cholangiocarcinoma.^60^ The GABA_A_R_Q_ and GABA_A_R_A3_ subunits of GABA_A_R, which mediate inhibitory synaptic transmission in the CNS, were overexpressed in HCC tissues compared with adjacent nontumor liver tissues, and the knockdown of GABA_A_R_Q_ and GABA_A_R_A3_ expression in malignant hepatocytes resulted in attenuated tumor growth both in vitro and in vivo.^17^, ^61^ In contrast, GABA_A_R_B3_ exerts an opposite function.^62^ The overexpression of GABA_A_R_A3_ also promoted the migration, invasion, and metastasis of breast cancer cells.^68^ GABA_A_R_P_, a subunit of GABA_A_R, has been reported to promote tumorigenesis, proliferation, and migration of PDAC cells, basal‐like breast cancer, ovarian cancer, and OSCC,^30^, ^55^, ^56^, ^67^ which confirms the function of GABA_A_R_P_ in the initiation and progression of cancer. In human thyroid cancer, the expression of GABA receptors was higher in tumors than in normal thyroid tissue, and GABA_A_R appeared to play a vital role. GABA_A_R_B2_ was detected in the blood vessels of normal thyroid and thyroid tumors but not in thyroid cancer cells, suggesting that GABA signaling contributes to angiogenesis in thyroid cancer. GABA_A_R_A2_ was detected in metastasis‐derived but not in primary‐tumor‐derived cell lines, suggesting its possible role in the development of metastases.^69^ Although the research by Roberts documents the expression of the GABAergic system in human thyroid tumors, further functional studies are needed.

#### 3.1.3 The effect of GABA_B_ receptors on cancers

GABA_B_R plays a positive role in cancer cell invasion in breast cancer^65^ and prostate cancer^22^ and a negative role in breast cancer,^66^ colon carcinoma,^59^ NSCLC,^57^ HCC,^63^ and cholangiocarcinoma.^60^ GABA_B_R_1e_ is overexpressed in human breast cancer cell lines and tissues and promotes the malignancy of breast cancer cells both in vitro and in vivo.^65^ The expression of the GABA receptor gene phenotype is associated with tumorigenesis and the clinical prognosis of NSCLC. A high expression of GABA_B_R_2_ with a low expression of GABA_A_R_A3_ may predict a better outcome.^57^

The diverse roles of GABA and its receptors need further description, which would yield more insights into novel drugs for treating cancers by targeting the regulation of GABAergic signaling. Ongoing efforts to characterize the mechanisms of GABA_A_R and GABA_B_R in cancers have revealed the potential downstream pathways of GABA receptors.

### 3.2 The mechanisms of GABAergic signaling in cancer progression and metastasis

GABAergic signaling contributes to tumorigenesis in systemic organs, and growing evidence has elucidated the potential pathways of tumor progression and metastasis. Moreover, the GABAergic system has recently been shown to exert immunosuppressive effects by disrupting the functions of various peripheral immune cells (Figure 1).

#### 3.2.1 cAMP pathway

Cyclic adenosine monophosphate (cAMP) signaling can regulate the biological behavior, including proliferation, migration, invasion, and metabolism, of cancer cells. Notably, cAMP signaling can either promote or suppress tumors depending on the cell type and the specific environment.^70^ Current reports have reported that cAMP plays a pivotal role in the inhibitory effect of GABA on tumors. GABA can suppress the proliferation and migration of NSCLC, PAC, and cholangiocarcinoma cells via cAMP signaling.^57^, ^58^, ^60^ Similarly, GABA can also inhibit colon cancer and HCC migration mediated by GABA_B_R and intracellularly transduced by a decrease in the cAMP concentration.^19^, ^59^, ^63^ Cancer‐induced bone pain (CIBP) remains a major challenge in advanced cancer patients. Zhou et al^71^ first described that the downregulation of GABA_B_R contributed to the development and maintenance of CIBP, and the restoration of depleted GABA_B_R attenuated CIBP‐induced pain behaviors at least partially by inhibiting the cAMP signaling pathway. Moreover, baclofen significantly inhibited mechanical allodynia and ambulatory pain induced by CIBP in a dose‐dependent manner.

#### 3.2.2 EGFR pathway

The epithelial growth factor receptor (EGFR) family has been widely researched in pharmacology owing to its close association with malignant proliferation.^72^ GABA_A_R can be activated by 5α‐androstane‐3α,17β‐diol (3α‐diol) in prostate cancer cells to transform androgen‐dependent EGFR pathways for the progression of castration‐resistant prostate cancer.^73^ Moreover, Wu et al^54^ demonstrated that increased GABA_A_R_A1_ might participate in this tumor‐promoting action by activating EGFR and the downstream signaling molecule Src. GABA_B_R can also transactivate EGFR through a ligand‐dependent mechanism, thus promoting the migration and invasion of prostate cancer cells.^74^ Furthermore, the GABA_B_R agonist baclofen selectively causes multisite phosphorylation of tyrosine residues associated with EGFR ubiquitination.^74^ Further the research group^65^ describes that GABA_B_R_1e_ favors EGFR signaling by displacing phosphatase nonreceptor type 12 (PTPN12) to disrupt the interaction between EGFR and PTPN12, which in turn promotes the growth and invasion of breast cancer cells.

EGFR‐mediated carcinogenesis may function by continuously phosphorylating downstream signaling effectors, including phosphatidylinositol‐3‐kinase (PI3K), protein‐serine–threonine kinase (AKT), or mitogen‐activated protein kinase (MAPK) pathways.

#### 3.2.3 AKT pathway

AKT is a serine/threonine kinase that participates in the critical role of the PI3K signaling pathway, which regulates the survival, invasion, migration, and epithelial‐mesenchymal transition (EMT) of cells. The kinase activity of AKT is induced by various growth factors, including EGFR, while the translocation of AKT from the cytoplasm to the inner surface of the cell membrane, a key process of AKT phosphorylation, depends on PI3K‐generated phospholipids. GABA may trigger several signaling molecules upstream of AKT to influence carcinogenesis through AKT signaling. Although GABA_A_R has been shown to regulate EGFR activation in prostate cancer, this condition was not observed in breast cancer cells. The overexpression of GAB_A_R_A3_ promotes the migration and metastasis of breast cancer through AKT signaling.^68^ GAB_A_R_A3_ may mediate signaling molecules upstream of the AKT pathway, like PI3K, rather than EGFR. Conversely, GABA_B_R can mediate the EGFR‐AKT signaling pathway. Moreover, in breast cancer, GABA_B_R_1e_ can activate the EGFR‐AKT pathway to promote the malignancy of breast cancer cells both in vitro and in vivo.^65^ Similarly, GABA enhances the malignancy of human high‐grade chondrosarcoma by promoting AKT signaling.^75^

#### 3.2.4 MAPK/ERK pathway

Mitogen‐activated protein kinases (MAPKs) or extracellular signal‐related kinases (ERKs) are serine/threonine kinases that transform extracellular stimuli into intracellular signals controlling cell proliferation and differentiation, and their dysregulation induces tumorigenesis.^76^, ^77^ Elevated intracellular Ca^2+^, induced by GABA to influence neurogenesis and synaptogenesis, activates the small guanine nucleotide‐binding protein Ras and stimulates the MAPK cascade.^78^ GABA_A_R, particularly GABA_A_R_P_, contributes to the proliferative and aggressive action of advanced tumors by activating MAPK/ERK signaling. GABA stimulates tumor growth in PDAC, basal‐like breast cancer, and ovarian cancer through GABA_A_R_P_ by activating the MAPK/ERK cascade by increasing intracellular Ca^2+^ levels.^30^, ^55^, ^67^ Similarly, GABA_A_R_P_ promotes the proliferation of OSCC by activating the MAPK pathway.^56^ GABA can also promote the growth of human gastric cancer cells in an autocrine or paracrine approach through GABA_A_R, followed by the activation of MAPK/ERK and an increase in cyclin D1.^23^ Besides GABA_A_R, GABA_B_R can also facilitate the invasion and metastasis of breast cancer mediated by promoting the phosphorylation of ERK1/2 and subsequently increasing the expression of MMP‐2.^79^ The MAPK/ERK pathway also involves the tumor‐suppressive effect of GABA. GABA decreases the in vitro cholangiocarcinoma growth through protein kinase A‐ and D‐myo‐inositol‐1,4,5‐triphosphate/Ca^2+^‐dependent pathways followed by the downregulation of ERK‐1/2 phosphorylation and cAMP.^60^

#### 3.2.5 MMP pathway

Matrix metalloproteinases (MMPs) are critical to tumor invasion and metastasis, as they influence the shape, movement, growth, survival, and differentiation of cancer cells by mediating the cytoskeletal machinery and cell adhesion.^80^ The overexpression of GAD1 is closely correlated with the invasion and metastasis of OSCC by the activation of MMP7.^81^ GABA promotes the invasion of prostate and breast cancer cells through GABA_B_R, and the increasing activities of MMPs, especially MMP‐2 and MMP‐3, are involved in the mechanism underlying this function.^18^, ^79^ Moreover, the MMP inhibitor GM6001 can significantly decrease GABA‐induced migration,^18^ with MMPs also being involved in the inhibitory effect of GABA in cancers such as colon cancer. Nembutal, a GABA agonist, inhibits the primary growth and metastasis of colon cancer, as it can reduce MMP production.^19^

Owing to the involvement of a complex cascade of events in cancer progression and metastasis, the above signaling pathways are intricately intertwined rather than independent of each other. Existing evidence has demonstrated that the activation of GABA_B_R downregulates the phosphorylation of ERK1/2 via the cAMP/PKA‐dependent pathway^60^ and consequently promotes the production of MMPs.^79^ GABA_B_R can reduce the activity of adenylyl cyclase and enhance the production of cAMP due to its association with G_i_ and G_o._ ^82^ Moreover, EGFR transactivated by GABA_B_R induces the activation of ERK1/2 by a G_i/o_‐dependent mechanism that requires MMP‐modulated pro‐ligand shedding.^74^ Further details about the modulatory mechanism need further clarification.

### 3.3 Cancer microenvironment

The GABAergic system is highly expressed in peripheral organs, and its control over the biological behavior of cells is also widespread through peripheral organs not only limited the CNS. The proliferation and metastasis of tumors benefit from GABA synthesized by not only cancer cells but also from the tumor microenvironment. Young et al^83^ reviewed the similarity of the brain and stem cell niches of peripheral organs and reported that GABAergic components were expressed in both normal and tumor conditions in the brain and peripheral organs. Neman et al^84^ found that the breast‐to‐brain metastatic tissue and cells displayed a GABAergic phenotype similar to that of neuronal cells. The GABA components, including GABAAR, GABA transporter, GABA transaminase, parvalbumin, and reelin, are all highly expressed in the metastasis of breast cancer to the brain. This indicates that breast cancers may metastasize to the brain when they escape their normative genetic constraints to adapt and coinhabit the neural microenvironment. GABAergic signaling not only provides the biosynthetic energy source for cancer proliferation but also disturbs normal cell proliferation, resulting in abnormal proliferation. All these findings pave the way for the development of a potential therapeutic target that disturbs the cancer microenvironment to inhibit tumor growth and metastasis for improved prognosis.

### 3.4 Cancer immunometabolism

Recent studies in the field of cancer immunometabolism have demonstrated that the production and consumption of metabolic products by different immune cells in various stages of differentiation and activation influence antitumor immune potential.^85^, ^86^ These small metabolites produced during the differentiation and activation of immune cells have better evolutionary potential as communication molecules than agents of classical signaling regulated by cytoplasmic, membrane‐bound, or secreted proteins. They can use fewer cellular resources for faster synthesis and secretion. Much attention has focused on the metabolism of glucose, amino acids, and fatty acids, although little is known about the role of metabolite GABA.

A recent study by Zhang et al^87^ reported that GABA is synthesized and secreted by activated B cells and confirmed that it can increase the expression of IL‐10 receptors, promote the differentiation and survival of anti‐inflammatory macrophages, and regulate the antitumor responses of CD8^+^ T cells through GABA_A_R in a mouse model of colon cancer. Previous studies demonstrated that several immune cells express GABA_A_R, and the activation of GABA_A_R through GABA binding inhibits inflammation. The activation of GABA_A_R on T cells restrains inflammatory T cells, namely, helper CD4^+^ T cells and killer CD8^+^ T cells,^88^, ^89^, ^90^ while augmenting the numbers of regulatory T cells, which restricts inflammation.^12^, ^89^ Also, the activation of GABA_A_R on antigen‐presenting cells reduces their pro‐inflammatory properties.^91^, ^92^, ^93^ Zhang et al^87^ found that a GABA_A_R agonist named muscimol can inhibit the activation and proliferation of tumor‐infiltrating CD8^+^ T cells, whereas the GABA_A_R antagonist picrotoxin can promote the cytotoxic activity of CD8^+^ T cells and limit tumor growth in vivo. The use of picrotoxin paves a way for targeting metabolite signaling to boost the efficacy of immune checkpoint interaction and chimeric antigen receptor T‐cell (CAR‐T) therapies.

A new study by Huang et al^27^ more broadly reveals the molecular and functional mechanism of GABA in the inhibition of antitumor immunity. Rather than serving as metabolic fuel or building blocks, GABA derived from lung and colon cancers activates GABA_B_R to stabilize β‐catenin induced by the ectopic expression of GAD1. Thus, it suppresses the expression of CCL4 and CCL5 to prevent the recruitment of dendritic cells, subsequently promoting tumor progression in an autocrine manner and inhibiting antitumor immune cell infiltration in a paracrine manner.

This emerging work reinforces the need to annotate metabolites within the cancer microenvironment, as it may boost the development of targeted therapy for the inhibition of tumor growth and metastasis through immunomodulation and metabolites.

## 4 TARGETING GABA RECEPTORS FOR CANCER THERAPY

With growing evidence implicating the role of GABA receptors in tumor progression and metastasis, a novel strategy for cancer treatment by manipulating GABA receptors comes in view. Hence, GABA agonists and antagonists represent direct therapeutic strategies for cancer treatment (Table 2). The functional activity of GABA_A_R can be enhanced by GABA_A_R agonists, including benzodiazepines, barbiturates, zolpidem, and propofol, and can be attenuated by GABA_A_R antagonists, including bicuculline and flumazenil (which attach at the GABA_A_R recognition site) and picrotoxin (which attaches elsewhere on the receptor complex).^94^ Like GABAAR, the activity of GABA_B_R is also regulated by certain drugs. For instance, it can be activated by baclofen and competitively antagonized by phaclofen. A growing body of selective GABA_B_R agonists and antagonists has been developed, such as the agonist CGP44532 and the antagonists CGP55845, CGP54626, and CGP35348.

**TABLE 2:** The potential GABA agonists and antagonists as cancer therapy drugs.

Classification	Name	Target	Cancer	Effect	Researches
Agonists	Benzodiazepines	GABA_A_R	Medulloblastoma	Inhibitory	^109^
	Nembutal	GABA_A_R	Colon cancer	Inhibitory	^19^
	Propofol	GABA_A_R	Lung cancer, breast cancer	Promotive	98, 99
	Baclofen	GABA_B_R	Colon cancer, Hepatocellular carcinoma	Inhibitory	59, 96
			Breast cancer, prostate cancer	Promotive	^74^
	CGP7930	GABA_B_R	Prostate cancer	Promotive	^74^
Antagonists	Bicuculline	GABA_A_R	Pancreatic Cancer	Inhibitory	^30^
	Flumazenil	GABA_A_R	Metastatic neuroendocrine cancers	Inhibitory	^97^
	Picrotoxin	GABA_A_R	Prostate cancer	Inhibitory	^54^
	CGP55845	GABA_B_R	Breast cancer	Inhibitory	^79^
	CGP54626	GABA_B_R	Prostate cancer, high‐grade chondrosarcoma	Inhibitory	74, 75
	CGP35348	GABA_B_R	Prostate cancer	Inhibitory	^18^

Nembutal, a barbiturate, exerts agonistic effects on GABA and is a common anesthetic, hypnotic, and anticonvulsive drug.^95^ Thaker et al^19^ first confirmed that nembutal effectively inhibits both primary development and metastasis of colon cancer by promoting apoptosis in vivo. Further, Joseph et al^59^ used baclofen as a specific GABA_B_R agonist to inhibit the norepinephrine‐induced migration of colon carcinoma cells. Wang et al^96^ demonstrated that baclofen also suppresses HCC tumor cell growth both in vitro and in vivo, and these inhibitory effects can be abrogated by pretreatment with phaclofen. Baclofen may function by suppressing cell proliferation with G0/G1 phase arrest by inhibiting cyclic adenosine monophosphate (cAMP) and downregulating the protein level and phosphorylation of p21^WAF1^.^96^ In prostate cancer, although baclofen had no effect on tumor growth, GABA_A_R antagonists such as picrotoxin have demonstrated antitumor efficacy.^54^, ^73^ Similar anti‐proliferative effects have been obtained using GABA_A_R antagonists such as flumazenil and bicuculline in cancers.^30^, ^97^ GABA_B_R antagonists, including CGP55845, CGP54626, and CGP35348, can inhibit GABA_B_R‐induced migration.^18^, ^74^, ^75^, ^79^ Kanbara et al^75^ confirmed that these antitumor effects may be implemented via different signaling pathways, including Ca^2+^ channels, cell cycle arrest, and apoptosis in cancer cells.

Although GABA receptor agonists and antagonists have significant benefits on the behavior of tumors by suppressing the proliferation and metastasis of cancer cells, their dual effect on cancers cannot be ignored. Contrary to the inhibitory effect of baclofen (GABA_B_R agonist) in colon and liver cancers, baclofen can exert the opposite effect on other cancers. Baclofen significantly promoted cell invasion and migration in vitro and metastasis in vivo.^74^, ^79^ The GABA_A_R agonist propofol activates GABA_A_R to downregulate the expression of the tripartite motif (TRIM)21, consequently upregulating the expression of Src, a protein that regulates cell adhesion and extension, causing the potentiation of tumor metastasis in vivo.^98^ Similarly, breast carcinoma cells also responded to propofol with increased migration which was mediated by calcium influx and actin reorganization.^99^ As baclofen and propofol are both commonly used anesthetics in surgical tumor resection, their pro‐metastatic effects need careful deliberation for the surgeon.

These studies provide a novel‐targeted treatment of individuals using GABA receptor agonists or antagonists modulating GABA for the prevention of several cancers, as well as suggest clinical considerations for cancer metastasis. While several existing agonists and antagonists can selectively target GABA_A_R and GABA_B_R,^100^, ^101^, ^102^ it is vital to maximize the utilization of appropriate compositions to optimize antitumor efficacy, owing to the significantly varying expressions of different receptor subtypes on cancer cells. These studies are needed to facilitate the development of various structural types of GABA agonists or antagonists for the treatment of cancers.

## 5 CONCLUSIONS AND PERSPECTIVE

GABAergic agents significantly vary from cancer tissues to paired noncancerous tissues and exert regulatory effects on the biological characteristics of cancer cells. The distinct effects of GABA receptors on cancer progression in multiple tumor types depend on both receptor type and physiological context. In most cases, GABA regulates cancer cell proliferation through the GABA_A_R pathway but regulates cancer cell invasion through the GABA_B_R pathway. Moreover, the regulatory signaling downstream of GABA receptors includes cAMP, EGFR, AKT, MAPK/ERK, and MMP. Presumably, the disparate subunits of GABA receptors target their specific downstream signaling to modulate cancer progression. The function and mechanism of GABA_A_R_P_, a subunit of GABA, are thoroughly described in multiple cancers (including PDAC, breast cancer, ovarian cancer, and OSCC), which underscores the pivotal role of GABA_A_R in cancer progression, with more subunits needing similar elucidation. A normal human body can maintain a balance in the GABAergic system. However, the altered expression of GABAergic components in the peripheral organs indicated the control of the cancer microenvironment on the proliferation of normal and cancer cells. Studies by Zhang et al and Huang et al^27^ add insights into how GABAergic components influence the immune microenvironment and tumor progression. This provides evidence for a novel therapeutic strategy for the treatment of cancers by modulating the GABAergic system.

Given the widespread distribution of GABAergic neurons in the nervous system, the most common side effects of drugs associated with GABA receptors include impaired CNS functions, such as sedation, ataxia, motor incoordination, anterograde amnesia, and paradoxical excitement. For instance, existing GABA_A_R inhibitors such as picrotoxin and bicuculline can induce severe convulsions in vivo due to their effect on GABA_A_R in the CNS.^103^ Therefore, an important strategy to avoid the risk of severe adverse reactions involves the development of selective GABA receptor agonists and antagonists that are highly specific to the receptor subunits (e.g., GABA_A_R_P_) and cannot penetrate the blood–brain barrier. Anesthetic drugs such as baclofen and propofol have an indispensable effect on tumor growth and metastasis, which is related to GABA receptors. These findings will attract more attention to the correlation between anesthesia and cancer metastasis, facilitating better prognosis for patients undergoing tumor resection.

In conclusion, the expression and molecular functions of GABA and its receptors in various cancers suggest that the GABAergic system is a potential target for anticancer therapy. This review provides an avenue for the development of more selective drugs that manipulate the activity of GABA receptors.

## AUTHOR CONTRIBUTIONS

**Tianjiao Li:** Conceptualization (lead); data curation (equal); methodology (equal); project administration (equal); resources (equal); visualization (equal); writing – original draft (lead); writing – review and editing (lead). **Jian Jiang:** Conceptualization (equal); investigation (equal); methodology (equal); writing – original draft (supporting); writing – review and editing (supporting). **Yaling Tang:** Conceptualization (lead); formal analysis (equal); funding acquisition (lead); writing – review and editing (lead). **Xinhua Liang:** Conceptualization (lead); funding acquisition (lead); investigation (lead); resources (equal); writing – review and editing (lead).

## CONFLICT OF INTEREST STATEMENT

The authors have no conflict of interest to declare.

## Data Availability

Data availability is not applicable to this article as no new data were created or analyzed in this study.
